# 3D Space Layout Design of Holographic Command Cabin Information Display in Mixed Reality Environment Based on HoloLens 2

**DOI:** 10.3390/brainsci12080971

**Published:** 2022-07-23

**Authors:** Wei Wang, Xuefeng Hong, Sina Dang, Ning Xu, Jue Qu

**Affiliations:** Air and Missile Defense College, Air Force Engineering University, Xi’an 710051, China; xueyvshahe@126.com (W.W.); fengxh1998@163.com (X.H.); xn187329@163.com (N.X.); qujue402@sina.com (J.Q.)

**Keywords:** ergonomics, mixed reality, 3D space layout, visual features, EEG

## Abstract

After the command and control information of the command and control cabin is displayed in the form of mixed reality, the large amount of real-time information and static information contained in it will form a dynamic situation that changes all the time. This brings a great burden to the system operator’s cognition, decision-making and operation. In order to solve this problem, this paper studies the three-dimensional spatial layout of holographic command cabin information display in a mixed reality environment. A total of 15 people participated in the experiment, of which 10 were the subjects of the experiment and 5 were the staff of the auxiliary experiment. Ten subjects used the HoloLens 2 generation to conduct visual characteristics and cognitive load experiments and collected and analyzed the subjects’ task completion time, error rate, eye movement and EEG and subjective evaluation data. Through the analysis of experimental data, the laws of visual and cognitive features of three-dimensional space in a mixed reality environment can be obtained. This paper systematically explores the effects of three key attributes: depth distance, information layer number and target relative position depth distance of information distribution in a 3D space, on visual search performance and on cognitive load. The experimental results showed that the optimal depth distance range for information display in the mixed reality environment is: the best depth distance for operation interactions (0.6 m~1.0 m), the best depth distance for accurate identification (2.4 m~2.8 m) and the overall situational awareness best-in-class depth distance (3.4 m~3.6 m). Under a certain angle of view, the number of information layers in the space is as small as possible, and the number of information layers should not exceed five at most. The relative position depth distance between the information layers in space ranges from 0.2 m to 0.35 m. Based on this theory, information layout in a 3D space can achieve a faster and more accurate visual search in a mixed reality environment and effectively reduce the cognitive load.

## 1. Introduction

The command and control interface on the battlefield is a multi-task complex interface with huge real-time data [1]. The command and control interface contains a large amount of real-time information and static information, which will form a dynamic situation that changes all the time [2]. In emergencies, system operators have a large burden of cognitive, decision-making and operation tasks [3,4]. With the gradual increase of weapons and equipment and the improvement of the degree of informatization, the total amount of information in the command and control interface will continue to increase, which requires a more reasonable and high-quality interface display method and information layout method [5].

With the development of new computer display technology, mixed reality (hereinafter referred to as MR) technology is becoming more and more mature. MR has the characteristics of a holographic display, the superposition and fusion of virtual and real and real-time information updates [6,7,8]. It can realize innovative functions such as the multi-dimensional real-time display of battlefield situation information, virtual–real integration deduction in a combat process and multi-channel agile interactive control of equipment. It has important application values in equipment information display and control, battlefield command and deduction and simulation training [9]. MR has become the development trend of how the battlefield will be displayed in the future.

However, with the continuous improvement of the construction of the command and control cabin in the mixed reality environment and the deepening of MR immersion, the information that the command and control cabin needs to process in the MR environment is also increasing [10,11]. A large amount of information is mixed in the MR command cabin environment, which, to a certain extent, will increase the burden of information processing and cognitive load, which will lead to the fatigue of the commander in the MR environment [12,13]. A greater cognitive load and fatigue will inevitably make people more likely to make mistakes in operation and judgment. How to solve this problem is an indispensable part of the construction of a qualified mixed reality information environment.

In response to this problem, it is necessary to optimize and improve the information space display in the MR environment, improve the commander’s interaction efficiency and reduce the commander’s cognitive load and fatigue. Through the layout design of the information space of the whole MR environment, the mixed reality information environment is more in line with the cognitive characteristics of users, so that users can carry out natural and efficient human–computer interactions in the MR information environment [14]. The design and information construction of the entire mixed reality information space should be based on human physiological characteristics and cognitive laws [15]. Therefore, it is necessary to carry out research on people’s visual search characteristics and cognitive loads so as to realize the three-dimensional spatial layout of information space in the whole MR environment based on the visual and cognitive characteristics of people [16,17].

The rest of the research section of this paper is structured as follows. The second part analyzes the research status of the information layout in two-dimensional and three-dimensional spaces at home and abroad. The third part introduces the subject information and the experimental equipment used in this paper and outlines the experimental sequence of the paper. The fourth part explores the distance range of the best depth for information display in 3D space in a mixed reality environment. In the fifth part, the optimal number of information layers for information display in a three-dimensional space under a mixed environment is explored. In the sixth part, the optimal depth distance range of the relative positions between the information layers in the three-dimensional space in the mixed reality environment is explored. The seventh part analyzes the above experimental results and draws conclusions.

## 2. Literature Review

In the MR environment, how to arrange the information and models in the display interface is essentially how to allocate human cognitive resources in the three-dimensional space [18,19]. In the process of people receiving visual information, the less cognitive resources are transferred, the longer the cognitive resources are concentrated and the better the efficiency of the people receiving information [20]. The transfer and concentration of human cognitive resources depends on the information space in which cognition functions [21,22].

Attention is a very important part of human cognitive resources. In visual tasks about people, attention plays a role in multiple stages of task processing [15]. Existing papers have studied the relationship between attentional concentration and task demands in visual searches, and their findings have found that there is a very important connection between visual search and attention [23]. There is little research at home and abroad on whether human cognitive resources can be deployed along the third dimension. Neuropsychological studies show that the deployment of cognitive resources depends on the encoding mode of a 3D space in the human brain. When cognitive resources are transferred from the nearby spatial part to the distant spatial part, the human cognitive system needs to change the spatial representation and vice versa [24]. Findings of this type suggest that representations of spatial states can have an impact on human cognitive resources.

The results of Remington and Pierce’s research show that the effect of the lateral distance of the target on the center target is consistent [25]. Sperling and Weichselgartner found similar results and concluded that the transfer duration of cognitive resources is independent of the lateral distance and distractors [26]. This shows that the lateral distance of information in a three-dimensional space does not affect the human cognitive load and the completion of visual search tasks. On longitudinal distance, Marrara’s research results showed that cognitive resources can be transferred in the depth direction, which indicates that the depth attribute of information will have an impact on the target [27]. Based on these results, it can be concluded that the horizontal distance of information in space does not affect the allocation of human cognitive resources, but human cognitive resources will change with the change of the depth distance of information.

Enns and Rensink demonstrated that scene-based attributes improve the efficiency of a visual search, and these attributes directly affect the saliency of objects [28]. Specifically, Enns and Rensink’s research showed that adding depth information to objects can improve the search performance. Wang Shulu and Ming Hai et al. studied 3D display technology based on the characteristics of human vision; explored the visual characteristics of human eyes in depth perception, spatial resolution and time, brightness, color resolution and other aspects and analyzed the human vision effect of a free stereoscopic display and the human vision effect of an integrated imaging and holographic display. Based on the research results of the human eye’s visual cognitive process of a 3D display, it provides theoretical support for the realization of a high-efficiency and low-load 3D display in a mixed reality environment [29]. The above research results indicate that the depth attribute of information has a certain influence on the visual search and cognitive efficacy.

Through a search task, Nonie J. Finlayson found that the visual search performance is related to the relative depth position of the target and the number of depth planes [30]. His results showed that different numbers of depth information planes have an impact on the visual search efficiency in a 3D search and that using depth information segmentation on the space shows that it may improve the performance of a visual search [31]. Erickson and St. James found that when a person’s cognitive resources are spread out over a larger area, fewer resources are used for each location. That is, when cognitive resources are distributed over a larger area of the visual field, the processing efficiency of this area decreases accordingly [32]. Treisman studied two-dimensional spatial layouts and found that when displayed information was presented in more spatial groups, participants found targets more slowly than in fewer spatial groups [33]. The above studies showed that the more information layers there are in the space, the worse the visual search performance and the greater the cognitive load. However, such studies only explore the relationship between the number of information display layers in a two-dimensional space and the visual search performance and cognitive load. However, the relationship between the number of information layers and performance and cognitive load has not been studied in a three-dimensional space.

These studies are all about human visual characteristics and cognitive characteristics. On the basis of this research, the experiment of human vision and cognition is carried out, and the general laws of vision and cognition in the layout of a three-dimensional space are obtained so as to carry out the three-dimensional space layout. There have been some results investigating the nature of the 3D search environment and how a visual search is affected by the 3D space, but most of these studies were conducted in real-world settings. There is still little progress in the related research on a layout design in three-dimensional space based on visual and cognitive characteristics in a mixed reality environment [34]. This research is to systematically explore the impact of three key attributes of object distribution in a three-dimensional space on visual search performance and cognitive load and to expand from a qualitative description to a quantitative description. These key properties are depth distance, number of information layers and target depth relative position. This is the theoretical basis to design the 3D layout of the information space in the mixed reality environment to achieve a faster, accurate and low-load visual search.

## 3. Methods

Subjects: Experiments were conducted by 10 students from an engineering university. All the subjects were in good physical condition, aged 20–30 years (SD = 2.4), and the visual acuity test results were: uncorrected visual acuity or corrected visual acuity of 5.0, no polarized astigmatism, no color blindness and no color weakness.

**Figure 1:**
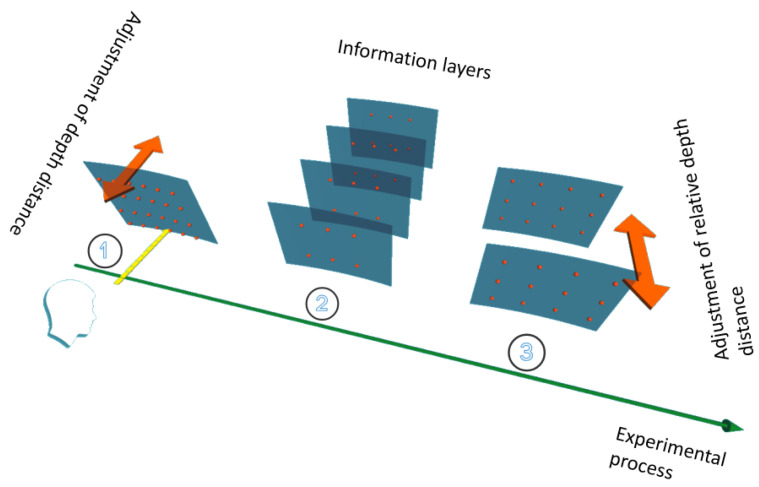
Schematic diagram of the experimental sequence.

Figure 1 is a schematic diagram of the experimental process. Experiment 1, Experiment 2 and Experiment 3 correspond to the depth distance experiment, the information layer number experiment and the relative depth distance experiment in the order of the arrows. In the experiment, the information target was placed in the blue translucent arc area to ensure that the subject’s sight and attention were focused on the information target area.

**Figure 2:**
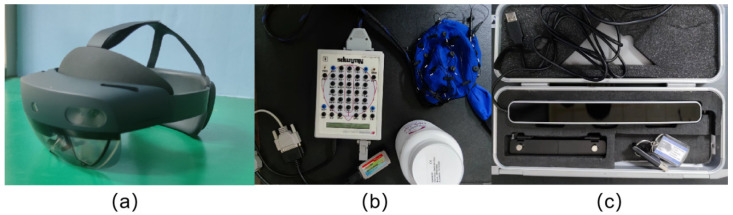
Experimental equipment. ((**a**) Mixed reality display: HoloLens2; (**b**) Electroencephalograph; (**c**) Eye tracker).

Experimental equipment: Microsoft’s HoloLens 2 was used for the construction of the mixed reality environment. As a typical mixed reality device, the device has good visual continuity and interactivity. Figure 2a is a real photo. The experimental test equipment includes an electroencephalograph and eye tracker. The electroencephalograph model is Nuamps7181; its analog input is 40-lead unipolar; the sampling frequency is 125, 250, 500 and 1000 Hz per lead; the input range is ±130 mV; the input impedance is not less than 80 MOhm; the common mode rejection ratio (CMRR) is 100 dB at 50/60 Hz; the input noise (Input Noise) is 0.7 uV RMS (4 uV peak-to-peak) and the interface is a USB interface and fully supports hot-swap technology. Figure 2b is a real picture. The eye tracker model is Tobii Pro Glasses 2, with a sampling rate of 50 HZ/100 HZ, which can automatically perform parallel parallax correction and has built-in slip compensation and tracking technology. The device also supports the absolute value measurement of the pupil, and its interface supports HDMI, USB and a 3.5-mm interface. Figure 2c is a real picture.

## 4. Depth Distance of Information Display in Mixed Reality Environment

### 4.1. Visual Sensitivity Experiment at Different Depth Distances in Mixed Reality Environment

The depth distance is defined as the *Z*-axis distance between the initial point and target information [35]. From the existing two-dimensional research on information layout, it can be concluded that the transfer of cognitive resources is independent of the lateral distance of the target [36], and the lateral movement of the human eye gaze point is also independent of the human eye movement characteristics [37]. At present, there is no research to prove whether the depth of the target information can affect people’s visual search and cognitive load, but the hypothesis is made through the above data analysis: Different depths of information in mixed reality environments lead to different sensitivities for people to perceive and receive information, verified by experiments.

**Figure 3:**
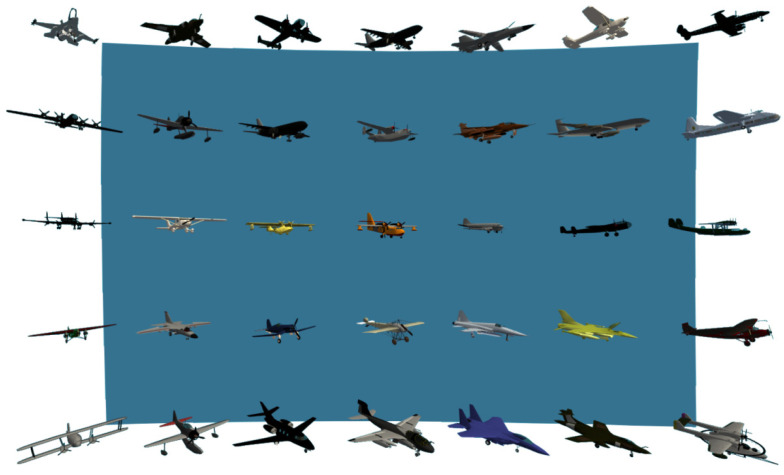
Information display layer in the visual sensitivity experiment.

The stimuli of the subjects were set to information of different depths in a mixed reality environment, and different types of aircraft were placed on the information display layer, as shown in Figure 3. In order to remove the influence of the visual model size change caused by the depth distance, when the depth of the information layer is deepened, the size of the information model on the information layer will be proportionally enlarged according to the deepening distance.

#### 4.1.1. Experiment Procedure

1. The subjects put on HoloLens 2 to enter the mixed reality environment and roughly browse the information in the environment.
2. The staff designates three aircraft of a specific type that need to be found in the entire information space as targets for the subjects, and the observation time is 30 s.
3. The subject finds all the targets in the information space and reports the number of rows and columns of the targets. The time requirement must be completed within 1 min. If the target is not found after the time limit, it will be handled as a search error.
4. Count the time and accuracy for the subjects to find the target.
5. Change the depth of the information display layer, replace the target, rearrange the position order of the models on the information surface and repeat the above experiment.
6. Analyze the time and accuracy information collected.

Before the experiment, it is necessary to determine the experimental depth distance range of the information layer. According to the practical requirements of command and control tasks and the limitations of the command and control environment, combined with the professional knowledge and experience of mixed reality human–computer interaction experts, combat command and control experts and actual operators, the requirements for the minimum depth distance of the information layer are put forward as follows: After the human arm is naturally raised, it can flexibly perform interactive actions such as clicking and dragging with the display layer to satisfy the shortest distance of the basic operation function of the display layer. and the subject’s first visual field includes a complete information display layer. This distance is determined to be 0.2 m. The maximum depth distance requirement of the information layer is as follows: the details of the information on the display layer can be clearly observed, and it is not larger than the radius of the command and control cabin. This distance is determined to be 5.0 m. The requirements for the interval of the depth distance of each group of the experiments are as follows: the experimental senses of the two groups of subjects have obvious differences. Group as many groups as possible within the closest and deepest distances.

In order to determine the interval requirements for the depth distance of each group of experiments, a pre-experiment is required before the main experiment. The experimental steps of the pre-experiment are the same as above, and the experimental data collected are the time to complete the task in each group of experiments. Paired *t*-tests were performed between the experimental data of each group and the experimental data of the first group of experiments to explore the smallest depth distance interval that had a significant effect on the subjects. In the pre-experiment, the initial depth distance of the information layer is 0.2 m, and the interval of each group of depth distances is 0.1 m.

#### 4.1.2. Data Cleaning Process

Before analyzing the experimental data, it is necessary to clean the experimental data to improve the quality of the experimental data and make the experimental results more accurate. The cleaning process of the experimental data is shown below.

Firstly, the overall data is analyzed, the data format is adjusted, the missing values and obviously wrong values in the data set are removed and the subjects corresponding to the missing values and obviously wrong values are re-experimented to complete the data. Second, the 3σ criterion is used to remove outliers in the data. Finally, the distribution of the processed data is detected, and the statistical chart of the data is drawn.

The experimental data of the pre-experiment:

It can be seen from Table 1 that, when the depth distance interval is 0.1 m, 0.2 m, 0.3 m and 0.4 m, *p* > 0.5, there is no significant difference. When the depth distance interval is 0.5 m, *p* < 0.05, there is a significant difference in the experimental data. The experimental results show that, when the depth distance interval is less than 0.5 m, the change of depth distance has no significant effect on the subjects, so the minimum depth distance interval of each group of experiments in the main experiment is 0.5 m.

#### 4.1.3. Main Experimental Data

Table 2 shows the time taken by the subjects to find three targets and the error rate of target finding at each depth distance.

One-way analysis of variance was performed on the time spent by the 10 groups of depth distances from the subjects, and the results showed that there were significant differences in the time spent by the subjects at different depth distances in the 10 groups (F(10,90) = 71.282, *p* < 0.001). It shows that different depth distances have a significant impact on visual tasks in mixed reality environments.

Calculate the index weights of the time and error rate and perform weighted fusion of the two sets of variables. The information weight method is used as the method for establishing the weights this time. The information weight method is a method to determine the index weights based on the information contained in the index data. This method determines the weights according to the discriminative information contained in the evaluation index [38]. Using the coefficient of variation method, the larger the coefficient of variation (CV), the greater the weight assigned, and the determination of the coefficient of variation is shown in Equation (1).

(1) $$CV=\frac{x_{i}}{s_{i}}$$

Among them:

$x_{i}$ is the average number of system indicators $X_{i}$;
$s_{i}$ is the variance of system index $X_{i}$.

Finally, the CV of time and accuracy is normalized, and the resulting value is the weight of these two indicators. The result is that the time weight is 0.1666, and the error rate weight is 0.8334.

**Figure 4:**
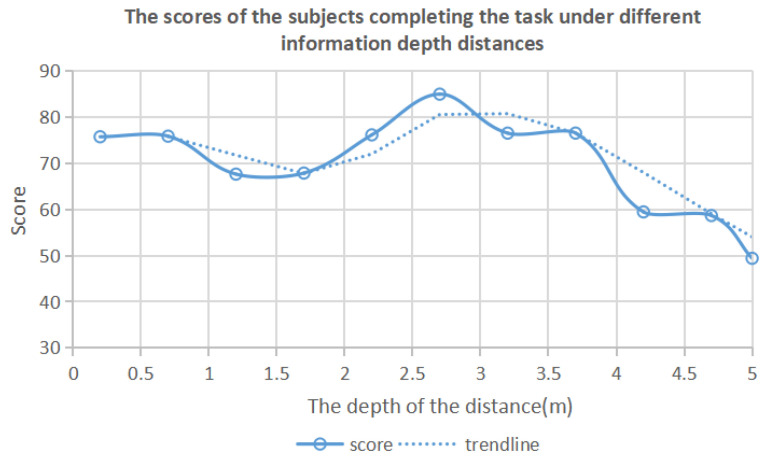
The trend graph of the scores of the subjects completing the task at different depth distances.

After weighted fusion of the time and error rate data, the resulting number is defined as the fusion number. In order to more effectively and intuitively observe and analyze the positive correlation between the depth distance of information and the observation target of the subjects, the score = 100 − fusion number is used to represent the analysis data of the depth distance of the information, as shown in Table 3 and Figure 4.

According to the analysis of Table 3 and Figure 4, when the depth distance of the information layer is between 0.2 m and 1.7 m, the experimental score decreases slowly. When the depth distance of the information layer is 1.7 m~2.7 m, the experimental score rises steadily and reaches the highest score when the depth distance is 2.7 m. When the depth distance of the information layer is between 2.7 m and 3.7 m, the experimental score begins to decrease. After the depth distance exceeds 3.7 m, the experimental score begins to decrease rapidly.

The experimental results of this experiment show that different depths of information in a space will affect people’s ability to perceive and receive information, and people’s sensitivity to information will change with the distance of the information depth. When the depth distance of the information layer is 0.2–3.7 m, the experimental score is maintained at a high level. Within this depth distance, the subjects have high information sensitivity and a strong ability to accept information. Therefore, in this experiment, a preliminary conclusion can be drawn from the change trend of the information sensitivity of the subjects: the information depth distance range in the three-dimensional information space in the mixed reality environment should be controlled between 0.2 m and 3.7 m.

Air defense command and control experts can divide the information display in the whole space into operation interactions, accurate identification and overall situation awareness based on the actual task requirements of the air defense information display. Among them, the operational interaction information has the characteristics of strong operability and frequent interactive actions. The depth distance of such information should be convenient for operators to operate on this type of information, and the visual search performance and cognitive resource concentration should be good. Accurately identifying information has strong requirements for visual search accuracy and a high concentration of attention, which requires that such an information depth should be arranged in the place where the visual search efficiency and cognitive resource concentration are the highest in the entire space. The overall situational awareness information has the characteristics of a large amount of displayed information and specific information presentation but does not require the timeliness of information. Therefore, this kind of information needs to be located at a depth distance with a wide field of view and does not require a high visual search efficiency and cognitive resource concentration.

By analyzing the above research data, combined with the characteristics and needs of the operating interactive information, the depth distance of such information must satisfy the operator that they can still interact with the information in the information space without changing their central position; therefore, the depth distance of such information is initially arranged in the range of 0.2 m~1.7 m. The accurate identification information needs to be arranged in the position with the best operator information sensitivity. Combined with the above information sensitivity experiment, the depth distance of such information is initially arranged in the range of 2.2 m~3.2 m, and the closer the distance is to 2.7 m, the better the effect is. For the overall situational awareness information, because this type of information has low requirements in timeliness and accuracy but has high visual field requirements, the depth distance of this type of information is initially arranged in the range of 3.2–3.7 m.

### 4.2. Determination of Depth Distance of Interactive Information

Through the above information sensitivity experiments, the rough depth distance range of operation interaction information is determined to be within 0.2 m~1.7 m. Based on this depth distance range, targeted experiments are conducted on the depth distance of the operation interaction information to refine the depth distance range of this kind of information combined with the visual cognitive load of the human to determine the most appropriate range of depth distance of such information. Since the minimum distance of the depth direction of the information model in the command and control interface is 20 cm, the depth distance of operation information is divided into 6 groups, with an interval of 20 cm ranging from 0.2 m to 1.7 m.

In the eye movement index, the blinking frequency can be used to show the degree of visual fatigue, the first fixation time can reflect the interest of the experimenter and the pupil diameter can represent the cognitive load level of the experimenter [39]. Since the experimenter cannot wear the HoloLens 2 and the eye tracker at the same time under the MR display interaction system, this experiment adopts the solutions proposed by Hirota, Masakazu and Kanda, Hiroyuki: Using the demonstration screen casting function in HoloLens 2, the experimenter and the tester are separated. The experimenter performs real-time screen projection from the first perspective in the process of completing the task. The tester cooperates with the experimenter to watch the first perspective of the experimenter completing the task. The eye movement data of the testers were collected using an eye tracker. Although the experimental data of different people will be different, the laws of visual fatigue and visual cognitive load characteristics of people are consistent, so the data obtained by this experimental method is valid [40].

In the design of the experimental process, according to the characteristics of the operating interactive information, the interaction task between the subject and the interface is added to the experimental task. The whole experimental design is as follows.

#### Experimental Design

7. The subjects put on HoloLens 2 to enter the mixed reality environment and roughly browse the information in the environment.
8. The staff assigned three aircraft of specific models to the subject as the target object, and the subject observed the target objects for 30 s.
9. The subjects found all the targets in the information space, clicked to select the target to change the color of the target and exchanged the position of the target with the position of other nontargets in the information space according to the requirements of the staff.
10. The time for the subjects to complete the task was counted, and the eye movement data of the subjects when they completed the task were collected.
11. Change the depth of the information display layer, replace the target, rearrange the position order of the model on the information layer and repeat the above experiment.

**Figure 5:**
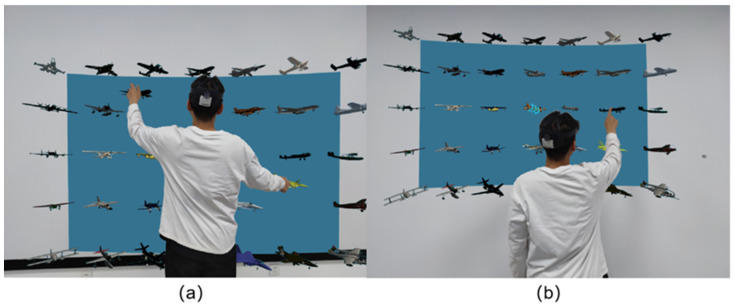
Operation process diagram in the depth distance experiment of manipulating interactive information ((**a**) Change the position of the target. (**b**) Change the color of the target).

During the experiment, in order to reduce the influence of the model size of the information on the experimental data, the information model and interface will be scaled according to the proportion when adjusting the depth distance. At the same time, the subjects were required to keep their feet in the initial position as much as possible during the operation. If they had to leave, they should return to the initial position immediately after completing the operation before the next operation was allowed. The interaction process of the subject’s operation is shown in Figure 5.

The collected task completion time and eye movement data were analyzed [41,42].

**Figure 6:**
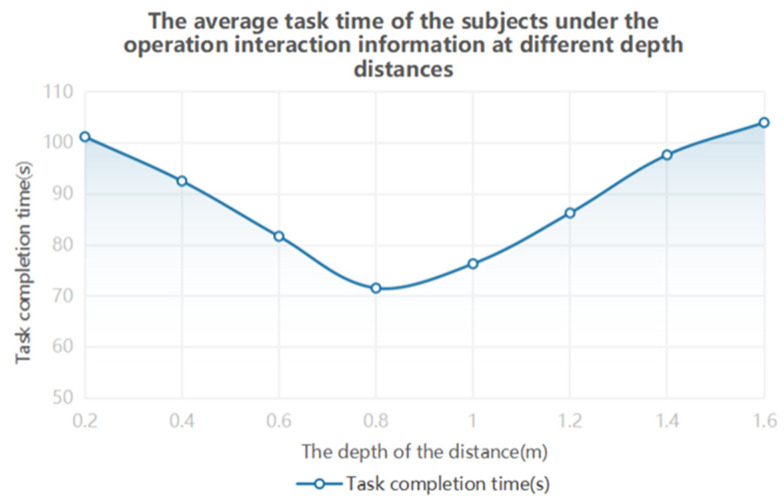
Time variation trend of operation interaction information to complete tasks at different depths and distances.

**Figure 7:**
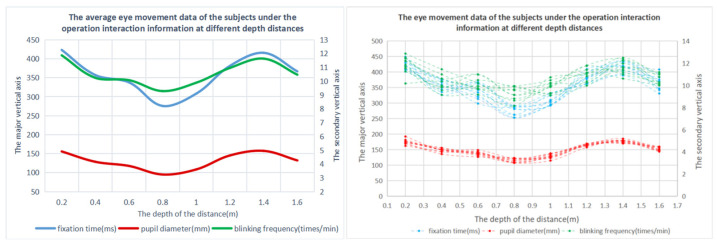
Variation trend chart of eye movement data of operation interaction information at different depths and distances.

One-way ANOVA results showed that gaze time, pupil diameter and blink frequency were significantly different at different depth distances. It is generally believed that fixation time, pupil diameter and blink frequency in eye movement indicators can reflect the difficulty of the visual perception and cognitive load levels of subjects. The above experimental results show that different information depth distances have important effects on the subject’s visual perception and visual cognitive load. According to Table 4 and Figure 6, when the depth distance of information is between 0.4 m and 1.2 m, the task performance of the subjects is better, and when the depth distance is 0.6 m, the task performance is the best. From the data reflected in Table 5 and Figure 7, it can be concluded that, when the information depth distance is 0.6–1.0 m, the subject’s visual perception effect is the best, and the visual cognitive load and visual fatigue are the lowest.

Combined with time and accuracy data and eye movement data, it can be concluded that the optimal range of depth distance for operational information is about 0.6–1.0 m.

### 4.3. Determination of Depth Distance of Precise Identification Information

**Figure 8:**
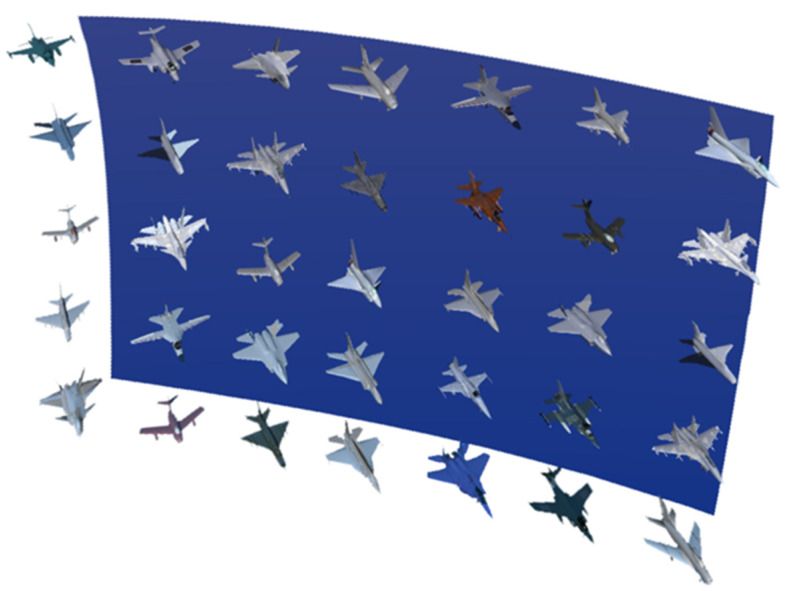
The information display layer in the accurate identification class information depth distance experiment.

The depth distance of accurate identification information determined by information sensitivity roughly ranges from 2.2 m to 3.2 m. Similarly, the depth distance of accurate identification information is divided into 6 groups with 20-cm intervals. Due to the characteristics of the accurate identification of information, the experimental targets were adjusted, and the original aircraft of different types and models were replaced with aircraft of the same type and different models, as shown in Figure 8. Since there is little difference in the styles of aircraft of the same type and different models, the use of this requires the subjects to have a higher grasp of the details of the information. Using this as an experimental target is more in line with the needs of the accurate identification of information.

#### Experiment Procedure

12. The subjects put on HoloLens 2 to enter the mixed reality environment and roughly browse the information in the environment.
13. The staff assigned three aircraft of specific models to the subject as the target object, and the subject observed the target object for 30 s.
14. The subjects found all the targets in the information space and reported the number of rows and columns of the target aircraft.
15. The time for the subjects to complete the task was counted, and the eye movement data of the subjects when they completed the task were collected.
16. Change the depth of the information display layer, replace the target, rearrange the position order of the models on the information surface and repeat the above experiments.

**Figure 9:**
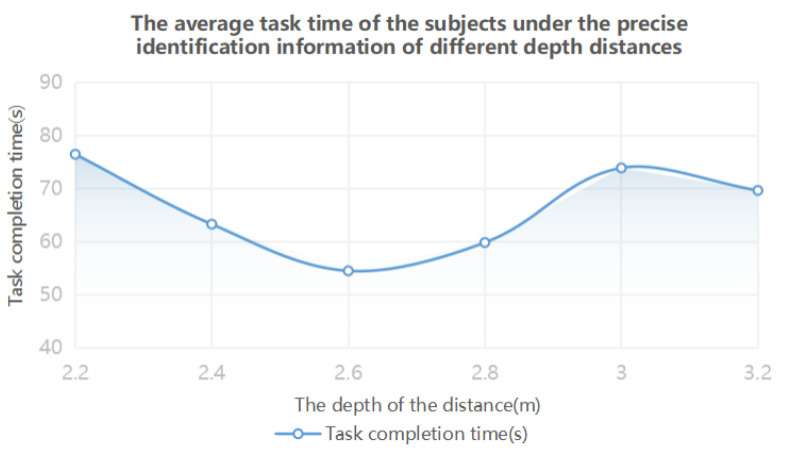
Time variation trend of the accurate identification information completing tasks at different depth distances.

**Figure 10:**
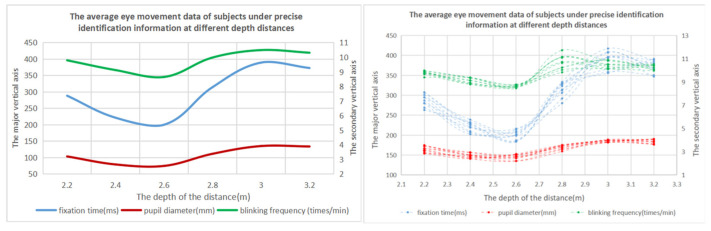
Variation trend diagram of eye movement data with different depths and distances of the accurate identification information.

The results of one-way ANOVA showed that the eye movement data were significantly different at different depth distances. From the analysis of Table 6 and Figure 9, it can be seen that when the depth distance of accurate identification information is 2.4–2.8 m, the performance of the subjects is the highest. The data in Table 7 and Figure 10 show that, when the depth distance of the accurate identification information layer is 2.2–2.8 m, the visual perception effect of the subjects is the best, and the visual cognitive load is the lowest.

Combined with the time and accuracy data and eye movement data, it can be concluded that the optimal range of the depth distance for the accurate identification information is about 2.4 m~2.8 m.

### 4.4. Determination of Depth Distance of Situational Awareness Information

The depth range of the situational awareness information determined by the information sensitivity experiment is 3.2 m~3.7 m. In the same way as the previous two types of information depth distances, the overall situational awareness information depth distances are divided into three groups at 20-cm intervals.

**Figure 11:**
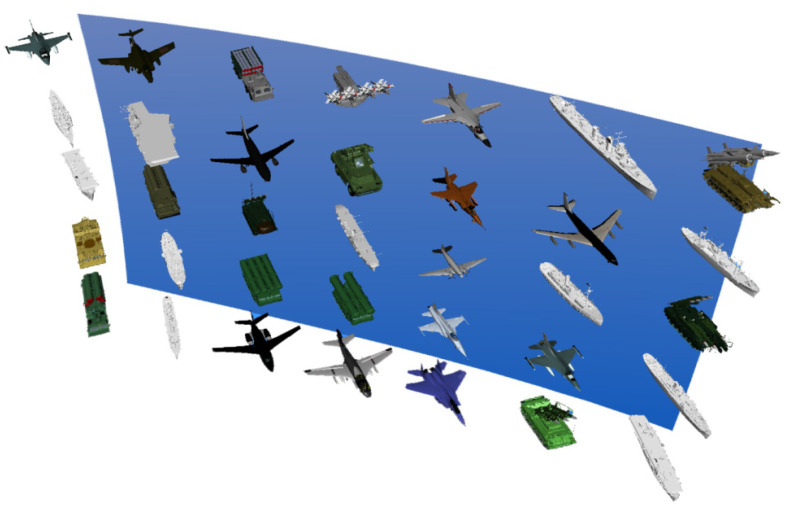
Information display layer of the depth distance experiment of situational awareness information.

According to the characteristics of the overall situational awareness information, the experimental stimuli were adjusted, and the original information containing only aircraft was replaced with information containing different numbers of aircraft, ships and Soviet anti-aircraft and anti-missile weapons, as shown in Figure 11.

#### Experiment Procedure

1. The subjects put on HoloLens 2 and entered the mixed reality environment.
2. Subjects observed the number of aircraft, ships and Russian air defense weapons in the entire information space.
3. After the subjects observed in the information space, they reported to the staff the number of aircraft, ships and Russian air defense weapons.
4. The observation time of the subjects to complete the experimental task was counted, and the eye movement data of the subjects when they completed the task were collected.
5. Change the depth of the information display layer, replace the target, readjust the number of different models on the information surface and repeat the above experiment.

**Figure 12:**
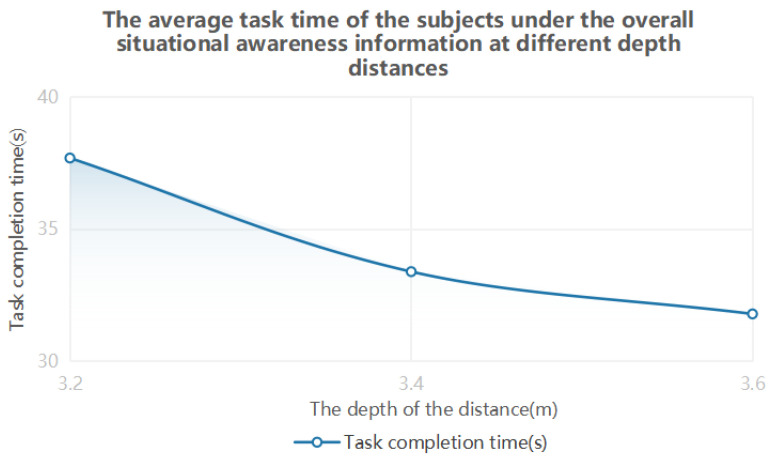
Time variation trend of the overall situational awareness information to complete tasks at different depths and distances.

**Figure 13:**
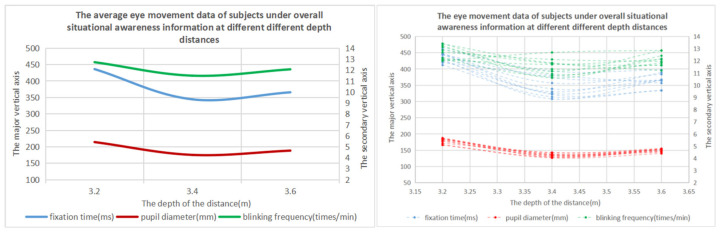
Change trend diagram of the eye movement data of the overall situational awareness information at different depths and distances.

The results of one-way ANOVA showed that the eye movement data were significantly different at different depth distances. It can be seen from Table 8 and Figure 12 that, when the depth distance range of the overall situational awareness information is 3.4–3.6 m, the subjects have the best performance in completing the task. It can be seen from Table 9 and Figure 13 that, when the overall situational awareness information depth range is 3.4–3.6 m, the subject’s visual cognitive load is the lowest, and the visual perception effect is the best.

Combined with the time and accuracy data and eye movement data, it can be concluded that the optimal range of the overall situational awareness information depth distance is about 3.4 m~3.6 m.

## 5. The Number of Layers of Information Displayed in Mixed Reality Environment

In previous studies, it has been explored that an increase in the number of information planes in a two-dimensional display environment will cause subjects to find the target information slower. The relationship between information search efficiency and cognitive load and the number of information display layers in 3D have not been studied. This experiment aims to obtain the relationship between information search efficiency, cognitive load level and information display layer number in the three-dimensional case, so as to obtain the range of the optimal information layer number in a visual direction.

**Figure 14:**
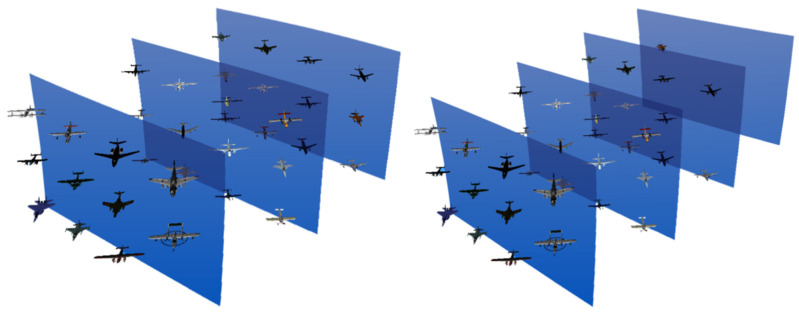
Experimental diagram of the information layers.

The tested targets were set as different numbers of information display layers in the mixed reality environment, and different types of aircraft were placed on the information display layers, as shown in Figure 14. In order to remove the influence of the amount of information on the experiment, when the number of display information layers is increased, the total amount of information in the entire mixed reality information space remains unchanged.

### 5.1. Experiment Procedure

1. The subjects put on HoloLens 2 to enter the mixed reality environment and roughly browse the information in the environment.
2. The staff assigned three aircraft of specific models to the subject as the target objects, and the subject observed the target object for 30 s.
3. The subjects found all the targets in the information space and reported the number of rows and columns of the targets. The time requirement must be completed within 1 min. If the time exceeded, the found targets were treated as search errors.
4. The time and accuracy of the subjects finding the target were counted, and the EEG and eye movement data of the subjects when they were looking for the target were collected.
5. Increase the number of information display layers, replace the target, rearrange the order of the positions of the models on the information layer and repeat the above experiments.
6. The time and accuracy information collected, as well as the EEG and eye movement data, were analyzed.

From the experimental results of the visual sensitivity experiment, it can be seen that, when the depth distance between the subject and the information layer is 2.6 m, the visual search effect is the best, and the cognitive resources are the most concentrated. As shown in Figure 4, according to the scores of the subjects’ information sensitivity when the information layers are at different depth distances, the number of information layers and the matching method of the distances in this experiment are determined. That is, try to place the information layer in the information space in the position where the visual search effect and the concentration of cognitive resources are the best for the subjects. The specific placement method is shown in Table 10.

### 5.2. Experimental Data

One-way analysis of variance was performed on the time spent by the subjects in the above seven groups of information layers. The results showed that there were significant differences in the time spent by the subjects under different depth distances in the 10 groups (F(6,63) = 104.212, *p* < 0.001). It shows that the number of different depth information layers has a significant impact on the visual tasks in a mixed reality environment.

**Figure 15:**
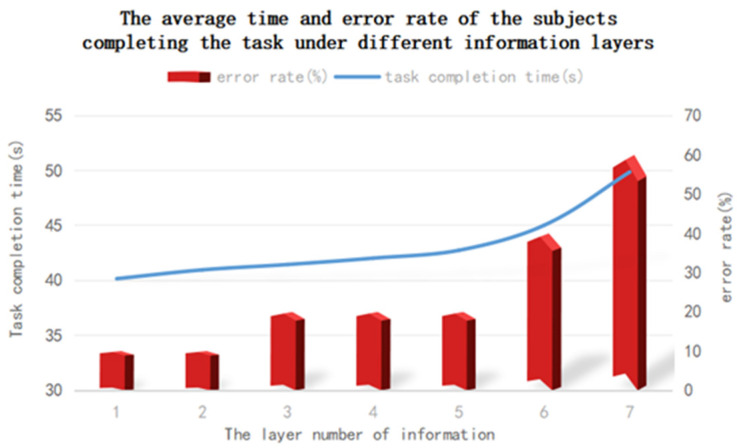
Time and error rate change trends of subjects completing tasks under different numbers of information layers.

From the analysis of the data in Table 11 and Figure 15, it can be seen that, with the continuous increase in the number of information layers, the time taken by the subjects to complete the task also increased, and the probability of the subjects making mistakes also increased. When the number of information layers in the information space is less than five, the subjects’ task completion time and error rate increase, but the increase is relatively slow. When the number of information layers in the information space is more than five, the subjects’ task completion time and error rate increase rapidly and have a clear growth trend.

The average eye movement data of the subjects when they completed the task were collected, as shown in Table 12.

**Figure 16:**
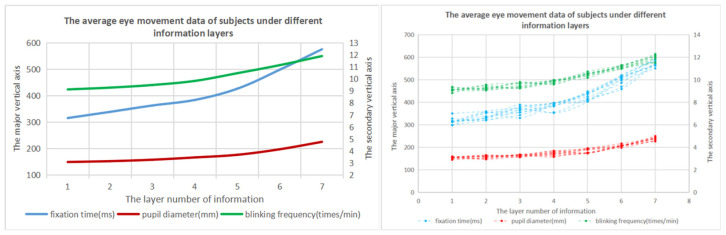
Change trend graph of subjects’ eye movement data under different numbers of information layers.

One-way ANOVA was performed on the time spent by the subjects in the above seven groups of information layers. The analysis results show that there are significant differences in the eye movement data of the subjects under different numbers of information layers. It can be seen from Table 12 and Figure 16 that, when the number of information layers in the information space increases, the subject’s gaze time, pupil diameter and blink frequency also increase when they complete the task. When the number of layers is less than five layers, the eye movement data of the subjects increases slowly, and when the number of information layers exceeds five layers, the eye movement data of the subjects increases more obviously.

According to the above data, when the number of information layers in the information space is one to five, the subject’s visual fatigue and visual cognitive load are in a state of slow growth. When the number of information layers is between five and seven, the visual fatigue and cognitive load increase significantly, and the increasing trend is obvious.

The brain controls people’s cognitive activities, and brain activity can be observed through electroencephalography (EEG). Relevant information of human cognitive and psychological activities can be obtained from the data such as latency and the waveform of event-related potential (ERP) components, thereby providing guidance for the design of three-dimensional spatial layout in a deep level [43]. The P300 component is a downward-positive peak that appears approximately 300 ms after an event (e.g., visual and auditory stimuli). The study found that the amplitude of the P300 component was positively correlated with the cognitive resources invested, and the greater the difficulty of the task, the longer the incubation period of the P300 peak [44].

**Figure 17:**
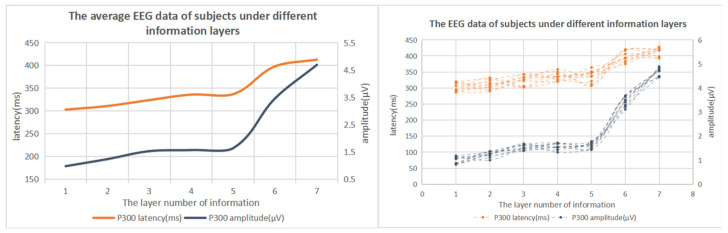
Variation trend of the P300 amplitude and latency of subjects under different numbers of information layers.

In the experiment of the number of information layers, the average EEG data of the subjects completing the task were collected, as shown in Table 13 and Figure 17.

The results of one-way variance showed that the EEG data had significant differences under different numbers of information layers. The analysis of Figure 17 and Table 12 showed that, with the increase in the number of information layers in the experiment, the latency and amplitude of the subjects’ P300 also increased. However, when the number of information layers is less than five, the growth of EEG data is relatively slow, and when the number of information layers exceeds five layers, the EEG data of the subjects increases significantly.

Through the above data analysis, it can be seen that, with the increase of the number of information layers in the information space, the cognitive load of the subjects also increases gradually. When the number of information layers in the information space is less than five, the cognitive load of the subjects increases slowly. When the number of information layers exceeds five layers, the cognitive load of the subjects has a clear upward trend, and the cognitive load is significantly improved.

### 5.3. Objective Experimental Data Analysis

Comprehensive analysis of objective data such as time usage, error rate, eye movement and EEG of different information layers yields: The greater the number of information layers in the information space in the mixed reality environment, the lower the ability and efficiency of people to obtain information and the higher the cognitive load. When the number of information layers in the space is less than five, the subject’s time to complete the task, error rate, visual fatigue and cognitive load increase slowly, and the increase is not obvious; When the number of information layers in the information space is greater than five, the subject’s task completion time, error rate, task performance, visual fatigue and cognitive load increase rapidly, and there is a clear growth trend. It can be seen that, in the mixed reality environment, the number of information layers in the information space is equal to five, which is an obvious inflection point on the growth trend of the entire objective data.

### 5.4. Subjective Evaluation

Time, error rate, eye movement and EEG data do not reflect the subjective feelings of the subjects. Operators can quickly learn and make decisions when using the mixed reality interface and because behavioral decisions are based on personal knowledge and habits. Therefore, it is necessary to evaluate the subjective feelings of the subjects from multiple perspectives. The NASA-TLX cognitive load subjective evaluation method can accurately and multi-dimensionally carry out subjective evaluations and is also widely used [45]. Ten subjects filled in the NASA-TLX scale after completing the experimental task and investigated the number of information layers in the three-dimensional space based on the results of the subjects’ evaluation.

The NASA-TLX evaluation scale has six-dimensional questions. Each dimension corresponds to a scale, with the far left of the scale representing 0 points and the far right representing 100 points. At the same time, the subjects were required to compare the six dimensions in pairs to determine the importance of each dimension, and the subjects checked the dimensions they thought were more important, as shown in Table 14.

The NASA-TLX evaluation score is shown in Formula (2):

(2) $$W_{i}=\frac{N_{i}}{N},N=\sum_{i=1}^{6}N_{i}\;(i=1\sim 6)$$

where $N_{i}$ is the number of times that the *i*th dimension is selected as the important dimension by the subject.

(3) $$W=\overset{6}{\underset{i=1}{\sum }}W_{i}R_{i}$$

where $R_{i}$ is the score of the *i*th dimension, and $W_{i}$ is the weight of the *i*th dimension.

After calculations, the NASA-TLX evaluation scale scores of the 10 subjects with different numbers of information layers are shown in Table 15.

From the NASA-TLX scale of the subjects, it can be concluded that there are differences in the cognitive loads of the subjects with different numbers of information layers in the space. As the number of information layers increases, the subjective quantification score of the subject’s cognitive load is higher. This suggests that fewer layers of information in the space is more in line with human cognitive properties. As shown by the data in Table 15, the less the number of information layers in the three-dimensional space, the better the efficiency and visual cognition of receiving information. Unlike objective data, subjective data does not have obvious inflection points and large data fluctuations.

### 5.5. Subjective and Objective Data Analysis Results

In the information layout of three-dimensional space, the number of layers should be controlled to reduce the number of information layers in the three-dimensional space. Secondly, according to the changing trend of the objective data, such as task completion time, error rate, eye movement and EEG, with the number of information layers, it can be concluded that the number of information layers should not exceed five in one visual direction when a hierarchical layout of the three-dimensional spatial information is carried out.

## 6. Depth Distance of Relative Position of 3D Spatial Information Layer

The distance of the relative positions of the information layers refers to the relative depth positions between the two information layers. The exploration of the relative positions of the information layers in the three-dimensional space in the mixed reality environment has not been involved in previous studies. In the existing research, the relative position distance refers to the depth distance between a single information target and other information. Those studies used flat-panel displays as the information display method, and the conclusions drawn on the influence of the relative position depth of the target information on the visual search and visual cognition in the study of such problems were not perfect. For example, Reis used a display that can adjust the depth position to conduct an experiment on the relative depth distance of information. The experimental results found that, when the subjects searched for characteristic targets, the subjects found the target faster when the relative depth position of the information was close [46]. However, this experiment is not specific enough to quantify the relationship between the relative position distance of the information and the search time and does not consider the visual cognitive load and fatigue.

Therefore, the research on the depth distance between the relative positions of the three-dimensional spatial information layers in the mixed reality environment is not only qualitative but also quantitative. Combined with eye movement, EEG data and subjective data, the influence of the depth distance between the relative positions of the information layers on the cognitive load of the subjects was explored. Finally, the subjective and objective data were combined to obtain the optimal relative position depth distance range between the information layers.

It can be seen from the above that the information layer in a mixed reality environment is divided into three categories: operation interaction, precise identification and overall situational awareness. According to the characteristics of the mixed reality environment constructed by HoloLens 2, when the information in the MR is occluded in the depth direction, the interactive operation of the information is prone to problems such as false touch and operation failure. Therefore, the operation interaction information layer is not suitable for information layered processing. Therefore, the analysis process of the depth distance of the relative position of the information layer is mainly aimed at the accurate identification information layer and the overall situational awareness information layer.

**Figure 18:**
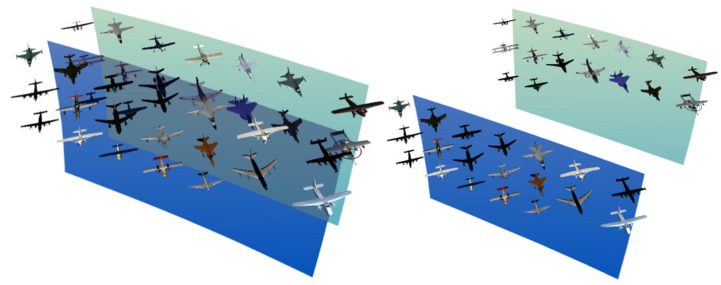
Depth distance experiment diagram of the relative position.

The experimental targets are two information display layers with different depths in a mixed reality environment, and different types of aircraft are placed on the two information display surfaces. The distance between the two information display layers can be adjusted, as shown in Figure 18.

### 6.1. Experiment Procedure

1. The subjects put on HoloLens 2 to enter the mixed reality environment and roughly browse the information in the environment.
2. The staff assigned three aircraft of specific model to the subject as the target object, and the subject observed the target object for 30 s.
3. The subjects found all the targets in the information space and reported the number of rows and columns of the targets. The time requirement must be completed within 1 min. If the time exceeded, the found targets were treated as search errors.
4. The time and accuracy of the subjects finding the target were counted, and the eye movement data, EEG data and subjective data were collected when the subjects were looking for the target.
5. Change the depth distance of the relative positions of the two information display layers, replace the target, rearrange the position order of the models on the information layer and repeat the above experiments.
6. The collected timing and accuracy information, eye movement and EEG data and subjective assessment data were analyzed.

The above experiments show that the optimal range of depth distance for accurate identification information is 2.4 m~2.8 m, and the optimal range of depth distance for overall situational awareness information is 3.4 m~3.6 m, so the depth distance of the maximum relative position is 0.4 m. The depth distance of the model radius in mixed reality is 0.1 m, so the depth distance of the minimum relative position is 0.1 m. In order to determine the intergroup interval of the relative position depth distance of each group in the experiment, a pre-experiment was performed before the experiment. The pre-experimental process is to continuously increase the depth distance of the relative position of the information layer from 0.1 m, each time increasing by 0.01 m. Collect the time for the subjects to complete the task in each group of experiments, and select the depth distance of the smallest relative position that has a significant difference from the first group of time data in each group of time data as the inter-group interval.

It can be seen from Table 16 that *p* > 0.05 when the relative position depth distance interval is 0.01 m, 0.02 m, 0.03 m and 0.04 m and *p* < 0.05 when the relative position depth distance interval is 0.05 m. It can be seen from the pre-experimental results that, when the interval between groups in the relative depth position experiment is greater than 0.05 m, there is a significant difference in the experimental data of the subjects. Therefore, the interval between groups in the main experiment was determined to be 0.05 m.

### 6.2. Main Experimental Data

Table 17, Table 18, Table 19 and Table 20 respectively show the performance data, eye movement data, EEG data and subjective scoring data measured in the experiment.

### 6.3. Data Normalization

Further statistical processing was performed on the measured subjective and objective data, and the results are shown in Table 21.

The matrix $X={\left(x_{ij}\right)}_{m\times n}$ is established to represent the data of the *j*th index under the *i*th scheme:

$$X={\left(x_{ij}\right)}_{m\times n}=\left[\begin{array}{ccc} x_{11} & \cdots & x_{1n}\\ \vdots & \ddots & \vdots \\ x_{m1} & \cdots & x_{mn} \end{array}\right]$$

Normalize the data:

$$x^{\prime }=x_{ij}^{\prime }=\{\begin{array}{c} \frac{x_{ij}}{\sum_{i=1}^{m}x_{ij}}\in \left[1,n\right],\;the\;bigger\;the\;better\\ \frac{1-x_{ij}^{\prime }}{\sum_{i=1}^{m}\left(1-x_{ij}^{\prime }\right)}\in \left[1,n\right],the\;smaller\;the\;better \end{array}$$

Get a normalized matrix: $x^{\prime }={\left(x_{ij}^{\;}\right)}_{m\times n}$

$$X^{\prime }={\left(x_{ij}{}^{\prime }\right)}_{m\times n}=\left[\begin{array}{ccc} x_{11}{}^{\prime } & \cdots & x_{1n}{}^{\prime }\\ \vdots & \ddots & \vdots \\ x_{m1}{}^{\prime } & \cdots & x_{mn}{}^{\prime } \end{array}\right]$$

$$0<x_{ij}{}^{\prime }<1,\;\overset{m}{\underset{i=1}{\sum }}x_{ij}{}^{\prime }=1\;$$

It can be obtained through the data belt matrix: $X={\left(x_{ij}\right)}_{7\times 8}$

$$X=\left[\begin{array}{c} \begin{array}{cccccccc} 47.395 & 0.2 & 417.58 & 4.18 & 10.25 & 3.46 & 367 & 59.44\\ 43.676 & 0.2 & 379.64 & 3.88 & 10.02 & 2.37 & 343 & 50.67\\ 45.397 & 0.1 & 357.89 & 3.58 & 9.48 & 1.53 & 315 & 45.33\\ 39.767 & 0.1 & 374.23 & 3.62 & 9.67 & 1.64 & 324 & 46.11\\ 43.064 & 0.1 & 380.09 & 3.53 & 9.56 & 1.24 & 319 & 45.56\\ 42.005 & 0.1 & 394.25 & 3.95 & 10.66 & 1.57 & 327 & 51.33\\ 44.839 & 0.2 & 423.15 & 4.27 & 11.65 & 3.26 & 356 & 54.89 \end{array} \end{array}\right]$$

Data Normalization: $X^{\prime }={\left(x_{ij}{}^{\;}\right)}_{7\times 8}$

$$X^{i}=\left[\begin{array}{cccccccc} 0.1409 & 0.1333 & 0.1411 & 0.1409 & 0.1427 & 0.1284 & 0.1406 & 0.1386\\ 0.1429 & 0.1333 & 0.1435 & 0.1427 & 0.1432 & 0.1405 & 0.1424 & 0.1428\\ 0.1420 & 0.1500 & 0.1448 & 0.1446 & 0.1445 & 0.1497 & 0.1443 & 0.1453\\ 0.1450 & 0.1500 & 0.1438 & 0.1443 & 0.1441 & 0.1485 & 0.1437 & 0.1449\\ 0.1432 & 0.1500 & 0.1434 & 0.1449 & 0.1443 & 0.1530 & 0.1441 & 0.1452\\ 0.1438 & 0.1500 & 0.1426 & 0.1423 & 0.1417 & 0.1493 & 0.1435 & 0.1425\\ 0.1423 & 0.1333 & 0.1408 & 0.1403 & 0.1394 & 0.1306 & 0.1414 & 0.1408 \end{array}\right]$$

Solve the weighted calculation of the normalized data:

$$y_{ij}=x_{ij}{}^{\prime }\cdot w_{i}\;$$

#### Determine the Evaluation Index Weight

The AHP hierarchical structure model was established by the AHP method [47]. A total of 12 experts participated in the establishment of evaluation index weights, all of whom have rich knowledge in human–machine efficacy, human–machine evaluations and mixed reality. After the statistics, analysis and calculations, the weight of each index was obtained, as shown in Table 22.

### 6.4. Quantitative Score

Sum the weighted scores of each indicator for the two display modes to calculate the weighted total score for each display mode:

$$T_{i}=\sum_{j=1}^{n}y_{ij}\;$$

Table 23 shows the specific values of the weighted total score of depth distance at different relative positions.

### 6.5. Experimental Data Analysis

**Figure 19:**
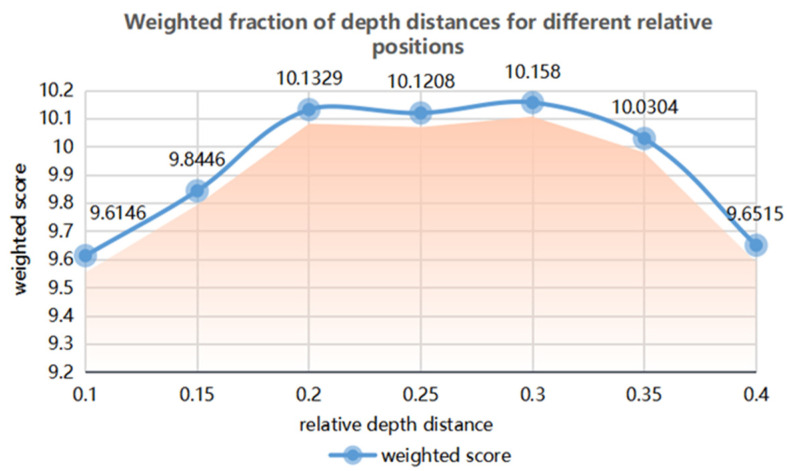
Trend of the weighted scores of the depth distances for different relative positions.

As shown in Figure 19, when the relative position distance of the information layer is 0.1–0.2 m, the scoring results of the comprehensive subjective and objective data have an upward trend. When the relative position distance of the information layer is 0.2–0.3 m, the scoring score of the comprehensive subjective and objective data reaches the highest, and the score is basically stable within this relative position range. When the relative position distance of the information layer is 0.3–0.4 m, the scoring score of the comprehensive subjective and objective data begins to decline.

The scoring results of the comprehensive task time, error rate, eye movement and EEG data and subjective scale data: the highest score is obtained when the relative position and depth distance of the information layer is between 0.2 m and 0.35 m. It can be seen that the depth distance of the relative position of the information layer in the three-dimensional space should be selected in the range of 0.2–0.35 m.

## 7. Conclusions

Based on the experimental research results of the depth distance of the information display, the number of information layers and the depth distance of the relative position of information under mixed reality, the following conclusions can be drawn:

1. According to expert experience and practical requirements and the experimental results of visual sensitivity, it can be known that the information in the 3D information space in the whole MR environment can be divided into three types of information: operation interaction, precise identification and overall situational awareness, according to the functional attributes. The optimal depth range of these three types of information is determined through the depth distance experiment, among which the optimal depth distance of the operation interaction type is 0.6 m~1.0 m; the optimal depth distance of the precise identification type is 2.4 m~2.8 m the overall situational awareness type is 3.4 m~3.6 m. The optimal depth distance is 3.4 m~3.6 m.
2. The less the number of information layers in the three-dimensional space in the MR environment, the better. However, during the actual command and control tasks, personnel need to process a large amount of information, and the types of information are complex and diverse. Therefore, it is necessary to perform hierarchical processing on the three-dimensional information space in the MR environment. According to the experimental results of the number of information layers in the MR environment, the number of information layers in a visual direction cannot exceed five layers.
3. The depth distance of the optimal relative positions between the information layers in the three-dimensional space in the MR environment ranges from 0.2 m to 0.35 m.

Conclusion 1 shows the optimal depth distance range of various types of information in a MR three-dimensional space. Conclusion 2 illustrates the arrangement principle of the number of information layers in a MR three-dimensional space. From conclusion 3, the depth distance of the optimal relative position between the information layers in the MR three-dimensional space can be obtained. A comprehensive analysis of all the above conclusions, the principle of the 3D spatial information layout of the holographic command cabin in the mixed reality environment can be obtained, which provides design suggestions for the construction of the 3D spatial information layout framework of the holographic command cabin. To a certain extent, it solves the problem that people’s burden in information processing increases and cognitive load increases when a large amount of information is mixed in the MR command cabin environment. It reduces the operational errors and judgment errors of the commander due to the confusion of information in the mixed reality space.

In reality, there is a certain blend of operational interactions, precise identification and overall situational awareness information. That is, some information has characteristics of two or more of the three types of information at the same time. This paper does not explore how to deal with such information with fusion properties. In future research, we will explore the display problem of fusion information and solve the three-dimensional spatial layout with fusion-attributable information by improving the interactive means. In this way, the limitations of this study are improved, and the layout design of the three-dimensional information space of the holographic command and control cabin in the mixed reality environment is enriched.

## Figures and Tables

**Table 1:** Significant differences in task completion time at different depth distance intervals.

Distance Interval (m)	0.1	0.2	0.3	0.4	0.5
**Significance**	t(9)=0.223 p=0.829	t(9)=0.360 p=0.728	t(9)=−0.016 p=0.988	t(9)=−0.211 p=0.838	t(9)=2.330 p=0.048

**Table 2:** The average time and error rate of the subjects to complete the task under different depth distances.

Distance (m)	0.2	0.7	1.2	1.7	2.2	2.7	3.2	3.7	4.2	4.7	5.0
**Time (s)**	45.56	44.79	44.18	42.79	43.09	40.13	40.85	40.78	43.29	48.02	53.64
**error rate (%)**	20	20	30	30	20	10	20	20	40	40	50

**Table 3:** The scores of the subjects completing the task under different depth distances.

Distance (m)	0.2	0.7	1.2	1.7	2.2	2.7	3.2	3.7	4.2	4.7	5.0
**Score**	75.74	75.87	67.64	67.87	76.15	84.98	76.53	76.54	59.45	58.66	49.39

**Table 4:** Average time to complete tasks after dividing the depth and distance range of operation interaction information.

**Distance (m)**	**0.2**	**0.4**	**0.6**	**0.8**	**1.0**	**1.2**	**1.4**	**1.6**	F(7,72)=19.225 p<0.001
**Time (s)**	101.23	92.55	81.71	71.56	76.33	86.28	97.67	104.02	

**Table 5:** Average eye movement data after dividing the depth and distance range of operation interaction information.

	Eye Movement	Fixation Time (ms)	Pupil Diameter (mm)	Blink Rate (Times/min)
Data Distance (m)				
0.2		423.11	4.91	11.87
0.4		356.87	4.16	10.24
0.6		337.62	3.86	10.07
0.8		275.63	3.24	9.28
1.0		308.21	3.61	9.89
1.2		381.22	4.62	10.96
1.4		415.51	4.95	11.63
1.6		366.56	4.26	10.47
ANOVA		F(7,72)=79.688p<0.001	F(7,72)=127.963p<0.001	F(7,72)=20.242p<0.001

**Table 6:** Time data after the depth distance range of the accurate identification information is divided.

**Distance (m)**	**2.2**	**2.4**	**2.6**	**2.8**	**3.0**	**3.2**	F(7,72)=9.792 p<0.001
**Time (s)**	76.45	63.25	54.43	59.78	73.87	69.62	

**Table 7:** Average eye movement data after the depth and distance range of the accurate identification information is divided.

	Eye Movement	Fixation Time (ms)	Pupil Diameter (mm)	Blink Rate (Times/min)
Data Distance (m)				
2.2		288.46	3.21	9.79
2.4		221.51	2.66	9.12
2.6		200.21	2.56	8.65
2.8		314.56	3.39	9.98
3.0		389.28	3.92	10.49
3.2		372.85	3.89	10.31
ANOVA		F(5,54)=229.969p<0.001	F(5,54)=88.513p<0.001	F(5,54)=102.657p<0.001

**Table 8:** Time data after division of the overall situational awareness information depths and distance ranges.

**Distance (m)**	**3.2**	**3.4**	**3.6**	F(2,27)=4.703p<0.05
**Time (s)**	37.7	33.4	31.8	

**Table 9:** The average eye movement data after dividing the overall situational awareness information depth and distance range.

	Eye Movement	Fixation Time (ms)	Pupil Diameter (mm)	Blink Rate (Times/min)
Data Distance (m)				
3.2		436.29	5.43	12.72
3.4		344.35	4.26	11.49
3.6		365.76	4.65	12.07
ANOVA		F(2,27)=44.162p<0.001	F(2,27)=123.834p<0.001	F(2,27)=11.052p<0.001

**Table 10:** The number of information layers and the depth distance of each information layer.

Number of Information Layers	The Depth Distance of Each Information Layer
1	No.1:2.7 m
2	No.1:2.2 m, No.2:3.2 m
3	No.1:2.2 m, No.2:2.7 m, No.3:3.2 m
4	No.1:2.2 m, No.2:2.7 m, No.3:3.2 m, No.4:3.7 m
5	No.1:1.7 m, No.2:2.2 m, No.3:2.7 m, No.4:3.2 m, No.5:3.7 m
6	No.1:1.2 m, No.2:1.7 m, No.3:2.2 m, No.4:2.7 m, No.5:3.2 m, No.6:3.7 m
7	No.1:1.2 m, No.2:1.7 m, No.3:2.2 m, No.4:2.7 m, No.5:3.2 m, No.6:3.7 m, No.7:4.2 m

**Table 11:** The average time and error rates of the subjects to complete the task under different numbers of information layers.

Number of Information Layers	1	2	3	4	5	6	7
**Time (s)**	40.158	40.959	41.449	42.025	42.739	45.022	49.863
**Error Rate (%)**	0.1	0.1	0.2	0.2	0.2	0.4	0.6

**Table 12:** The average eye movement data of subjects completing tasks under different information layers.

	Eye Movement	Fixation Time (ms)	Pupil Diameter (mm)	Blink Rate (Times/min)
Data Number of Layers				
1		315.23	3.07	9.12
2		338.66	3.14	9.27
3		362.74	3.26	9.49
4		383.80	3.45	9.83
5		426.27	3.67	10.47
6		497.95	4.13	11.14
7		575.68	4.75	11.90
ANOVA		F(6,63)=309.472p < 0.001	F(6,63)=204.538p < 0.001	F(6,63)=328.588p < 0.001

**Table 13:** Average P300 amplitude and average latency of subjects with different numbers of information layers.

	Number of Layers	1	2	3	4	5	6	7	ANOVA
EEG									
**P300 amplitude/** **μV**		0.98	1.24	1.53	1.57	1.64	3.46	4.69	F(2,27)=44.162p<0.001
**P300 latency/ms**		303	311	324	336	337	398	413	F(2,27)=44.162p<0.001

**Table 14:** NASA-TLX 6-dimensional comparison table.

Mental Demand Physical Demand	Mental Demand Temporal Demand	Mental Demand Performance Level	Mental Demand Effort Level	Mental Demand Degree of Frustration
Physical demand Temporal demand	Physical demand Performance level	Physical demand Effort level	Physical demand Degree of frustration	Temporal demand Performance level
Temporal demand Effort level	Temporal demand Degree of frustration	Performance level Effort level	Performance level Degree of frustration	Effort level Degree of frustration

**Table 15:** NASA-TLX average cognitive load assessment.

**Number of Layers**	**1**	**2**	**3**	**4**	**5**	**6**	**7**	F(6,63)=64.993p<0.001
**Average Score**	36.28	38.16	39.24	41.26	42.35	47.92	49.24	

**Table 16:** Significant differences in the task completion time at different relative position depth distance intervals.

Interval (m)	0.01	0.02	0.03	0.04	0.05
**Salience**	t(9)=−0.250 p=0.808	t(9)=0.363 p=0.725	t(9)=1.919 p=0.087	t(9)=2.238 p=0.052	t(9)=10.005 p=0.001

**Table 17:** The average time and error rate of completing the task by the depth distance of different relative positions of the information display layer.

**Relative Depth Distance (m)**	**0.1**	**0.15**	**0.2**	**0.25**	**0.3**	**0.35**	**0.4**	F(6,63)=26.591p<0.001
**Time (s)**	47.395	43.676	45.397	39.767	43.064	42.005	44.839	
**Error Rate (%)**	0.2	0.2	0.1	0.1	0.1	0.1	0.2	

**Table 18:** Depth distance of different relative positions of the information display layer, the average value of eye movement data for completing the task.

	**Eye Movement Data**	**Fixation Time (ms)**	**Pupil Diameter (mm)**	**Blink Rate (Times/min)**	F(2,18)=1826.4p<0.001
**Relative Depth Distance**					
0.1		417.58	4.18	10.25	
0.15		379.64	3.88	10.02	
0.2		357.89	3.58	9.48	
0.25		374.23	3.62	9.67	
0.3		380.09	3.53	9.56	
0.35		394.25	3.95	10.66	
0.4		423.15	4.27	11.65	

**Table 19:** The average value of the EEG data for different relative positions of the information display layer.

	Number of Layers	0.1	0.15	0.2	0.25	0.3	0.35	0.4
EEG								
**P300 amplitude/** **μV**		3.46	2.37	1.53	1.64	1.24	1.57	3.26
**P300 latency/ms**		367	343	315	324	319	327	356

**Table 20:** The average subjective cognitive load of the task completed by the depth distance of different relative positions of the information display layer.

**Relative Depth Distance (m)**	**0.1**	**0.15**	**0.2**	**0.25**	**0.3**	**0.35**	**0.4**	F(6,63)=34.871p<0.001
**Average Score**	59.44	50.67	45.33	46.11	45.56	51.33	54.89	

**Table 21:** Subjective and objective data at depth distances at different relative positions.

	Relative Depth Distance	0.1	0.15	0.2	0.25	0.3	0.35	0.4
Data Indicators								
**Task time**		47.395	43.676	45.397	39.767	43.064	42.005	44.839
**Error Rate**		0.2	0.2	0.1	0.1	0.1	0.1	0.2
**Fixation Time**		417.58	379.64	357.89	374.23	380.09	394.25	423.15
**Pupil Diameter**		4.18	3.88	3.58	3.62	3.53	3.95	4.27
**Blink Rate**		10.25	10.02	9.48	9.67	9.56	10.66	11.65
**P300 Amplitude**		3.46	2.37	1.53	1.64	1.24	1.57	3.26
**P300 Latency**		367	343	315	324	319	327	356
**Subjective Evaluation**		59.44	50.67	45.33	46.11	45.56	51.33	54.89

**Table 22:** Evaluation indicator weights.

Data Type	Task Time	Error Rate	Fixation Time	Pupil Diameter	Blink Rate	P300 Amplitude	P300 Latency	Subjective Evaluation
**Weights (%)**	9.929	10.706	5.810	10.211	13.537	11.688	11.688	26.431

**Table 23:** Weighted total score of the depth distance for different relative positions.

Relative Depth Distance (m)	0.1	0.15	0.2	0.25	0.3	0.35	0.4
Weighted Score × 70	9.6146	9.8446	10.1329	10.1208	10.1580	10.0304	9.6515

## Data Availability

The data presented in this study are available on request from the corresponding author. The data are not publicly available due to some data detailed equipment parameters; these parameters cannot be disclosed.
